# L’angioplastie du tronc commun non protégé de l’artère coronaire gauche: devenir des patients à court et moyen termes

**DOI:** 10.11604/pamj.2018.29.188.9640

**Published:** 2018-04-02

**Authors:** Yibar Kambiré

**Affiliations:** 1 Service de Médecine et Spécialités Médicales, Hôpital National Blaise Comparore, 11 BP 104 Ouagadougou, CMS 11, Burkina Faso

**Keywords:** Tronc commun coronaire gauche, angioplastie, stent, événements cardiovasculaires, Left main coronary artery, percutaneous intervention, stent, cardiovascular events

## Abstract

**Introduction:**

Notre but était de déterminer le devenir des patients traités par angioplastie du tronc commun non protégé de l'artère coronaire gauche.

**Méthodes:**

Une étude rétrospective a porté sur tous les patients traités par angioplastie du tronc commun non protégé de la coronaire gauche entre janvier 2004 et juin 2009. Les données cliniques et angiographiques ont été recueillies sur les dossiers médicaux, complétées par un entretien téléphonique avec le patient ou ses médecins.

**Résultats:**

Quarante-huit patients d'âge moyen de 68,50±14,06 ans ont été inclus. Les syndromes coronariens aigus étaient le principal motif d'admission (75%). Les lésions du tronc étaient distales (77,1%) et pluritronculaires (64,6%). Un stent nu a été implanté chez 73% des patients. Après un suivi moyen de 22 mois, le taux d'événements cardiovasculaires majeurs a été de 31,3% dont 20,8% de resténose et 14,6% de revascularisation. Le taux de décès hospitalier a été de 4,2%. Les facteurs prédictifs d'événements cardiovasculaires majeurs ont été un EuroSCORE ≥ 10 et les antécédents cardiovasculaires.

**Conclusion:**

L'angioplastie du tronc commun est une alternative dans les situations d'urgence ou en cas de contre-indication de la chirurgie. Le risque évolutif des patients est lié à leur risque global avant angioplastie.

## Abstract (en)

**Introduction:**

This study aimed to determine short and medium term outcomes in patients with unprotected left main coronary disease treated by percutaneous intervention.

**Methods:**

We conducted a retrospective study of all patients with unprotected left main coronary artery disease treated by percutaneous intervention between January 2004 and June 2009. Clinical and angiographic data were collected from their medical records and supplemented by a telephone interview with the patients or their doctors.

**Results:**

Forty eight patients with an average age of 68.50 ± 14.06 years were included in the study. Acute coronary syndromes were the primary reason for admission (75%). Patients had distal left main lesions (77.1%) and pluritroncular lesions (64.6%). Bare-metal stents were implanted in 73% of patients. After a mean follow-up of 22 months, the rate of major cardiovascular events was 35.4% (20.8% of restenosis and 14.6% of revascularizations). Hospital mortality rate was 4.2%. Predictive factors of major cardiovascular events included an EuroSCORE ≥ 10 and a history of cardiovascular disease.

**Conclusion:**

Angioplasty of the left main trunk is an alternative approach to emergency situations or when surgery is not indicated. Patients’ outcome is dependent on their overall risks factors before angioplasty.

## Introduction

La chirurgie de pontage aorto-coronarien est le traitement de référence des lésions du tronc commun coronaire gauche non protégé (TCGNP) car elle est mieux documentée et donne de meilleurs résultats par rapport au traitement médical seul [1, 2]. L'angioplastie est acceptée pour les patients non opérables ou avec gros risques opératoires [1, 2]. Les stents et particulièrement les stents actifs sont de plus en plus utilisés dans le traitement de ces lésions avec des résultats encourageants à court et moyen terme [3, 4]. La place de l'angioplastie est évolutive avec le temps et les preuves apportées par les études. Notre étude a pour but d'évaluer le devenir à court et moyen terme des patients ayant bénéficié d'une angioplastie du TCGNP au CHU de Clermont-Ferrand et contribuer ainsi à l'ancrage de l'angioplastie dans le traitement des lésions du TGNP.

## Méthodes

C'est une étude rétrospective descriptive couvrant la période du 1^er^ Janvier 2004 au 30 Juin 2009.

**Patients**: Tous les patients consécutifs âgés d'au moins 18 ans et ayant bénéficié d'une angioplastie du TCGNP dans le service durant la période de l'étude ont été inclus. Les angioplasties sur tronc commun protégé ont été exclues.

**Protocole d'angioplastie**: La voie artérielle fémorale ou radiale a été utilisée. Le choix et les caractéristiques du matériel technique étaient au choix du praticien. L'angioplastie a été encadrée par le conditionnement pharmacologique recommandé à la période de l'étude (double antiagrégant plaquettaire clopidogrel-aspirine avec dose de charge de clopidogrel 300 mg voire 600 mg et bolus intraveineux d'aspirine (250-500 mg) chez les patients qui n'étaient pas prétraités et un bolus intraveineux d'héparine non fractionnée de 30 UI/Kg en début de procédure renouvelée si l'angioplastie dure plus d'une heure. En cas de ponction radiale une administration intra-artérielle de vérapamil (1 mg) et de dinitrate d'isosorbide (1 mg) a été faite immédiatement après la ponction artérielle. Après la pose de stent la double antiagrégation plaquettaire Aspirine (75-160 mg/jour) et clopidogrel (75mg/jour) est poursuive pendant au moins un mois en cas de stent nu et pendant au moins 12 mois en cas de stent actif. Les anti-GPIIb/IIIa ont été utilisés au cas par cas en périprocédural. Une surveillance en unité de soins intensifs cardiologiques a été réalisée pour une durée variable selon le tableau clinique.

**Collecte des données**: Les données épidémiologiques, cliniques et paracliniques (échocardiographie doppler, électrocardiogramme, biologie et coronarographie) ont été recueillies sur les dossiers médicaux, et complétées par un entretien téléphonique avec le patient ou un membre de la famille, son médecin traitant et son cardiologue. Les données sur l'angioplastie ont concerné les caractéristiques des lésions coronaires, les indications et les résultats de l'angioplastie, les caractéristiques des stents, les événements cardiovasculaires enregistrés et leur traitement. Le succès angiographique a été défini par l'obtention d'un flux TIMI 3 en fin de procédure, une lésion résiduelle post-angioplastie ≤ 20% du diamètre du vaisseau traité, l'absence de décès, d'infarctus du myocarde ou de nécessité de revascularisation en urgence du vaisseau traité en fin de procédure. La resténose intrastent a été définie par une prolifération intrastent > 50% du diamètre du vaisseau. Le critère principal de jugement est un critère combiné appelé les événements cardiovasculaires majeurs (ECVM) définis par: décès toute cause, infarctus du myocarde avec onde Q, nécessité d'une revascularisation en urgence sur la lésion ou le vaisseau traité et la décompensation cardiaque. Le critère secondaire retenu a été la revascularisation sans urgence. En cas de survenue de plusieurs événements cardiovasculaires chez un même patient nous avons retenu en définitive l'événement le plus grave au terme du suivi. Les données recueillies ont été saisies et analysées par le logiciel EPI info 6.04. Les résultats ont été exprimés en moyenne ± écart type, en fréquence relative et en odds ratio. Le test de khi² et le test exact de Fischer ont été utilisés pour l'analyse statistique. Le seuil de signification statistique retenu a été de 5% pour un intervalle de confiance à 95%.

## Résultats

Sur 66 mois, 63 patients ont bénéficié d'une angioplastie du tronc commun de l'artère coronaire gauche dont 15 cas sur un tronc protégé. Notre étude a donc porté sur 48 patients. Il s'est agi de 58,3% d'hommes et 41,7% de femmes. L'âge moyen des patients était de 68,50 ± 14,06 ans (extrêmes 31 et 90 ans).

**Selon les facteurs de risque cardiovasculaire, antécédents et comorbidités**: Tous les patients avaient au moins un facteur de risque cardiovasculaire chacun; 70,8% d'entre eux en avaient au moins trois. Le nombre moyen de facteurs de risque par patient était de 3,25 ± 1,26 (extrêmes 1 et 5). Trente et trois patients (68,8%) avaient des antécédents ou des comorbidités dont 35,4% de coronaropathies. La répartition des patients selon les facteurs de risque cardiovasculaire, les antécédents et les comorbidités est présentée dans le Tableau 1.

**Tableau 1:** Facteurs de risque cardiovasculaire et antécédents de 48  patients traités par angioplastie du tronc commun non protégé de l’artère coronaire gauche

Facteurs de risque cardiovasculaire	Nombre de cas (n)	Pourcentage (%)
Age	39	81,3
HTA	28	58,3
Tabagisme*	26	54,2
Obésité/surpoids	25	52,1
Dyslipidémie	24	50,0
Diabète type II	9	18,8
Hérédité	7	14,6
Antécédents et co-morbidités**	33	68,8
Coronaropathies	17	35,4
Insuffisance cardiaque	9	18,8
Valvulopathie	6	12,5
Trouble du rythme ou de la conduction	6	12,5
Artériopathies périphériques	13	27,1
Accident vasculaire cérébral ou antécédents d’accident vasculaire cérébral	2	4,2
Maladie thromboembolique	2	4,2

**Clinique**: Les circonstances du diagnostic étaient majoritairement les syndromes coronariens aigus (SCA), 75% dont 25% avec complications hémodynamiques et/ou rythmiques. Les autres circonstances de découverte sont détaillées dans le Tableau 2. L'état clinique des patients était critique dans 20,8% des cas nécessitant l'administration d'amines vasopressives et/ou la mise en place d'une contre-pulsion par ballon intra-aortique. A l'admission 20,8% des patients avaient une insuffisance rénale.

**Tableau 2:** Tableau clinique ayant conduit au diagnostic de lésion du tronc commun non protégé de l’artère coronaire gauche chez 48 patients

Circonstances diagnostiques	Nombre de cas	Pourcentage (%)
Syndrome coronariens aigus sans sus-décalage de ST	22*	45,8
Syndrome coronariens aigus avec sus-décalage de ST	14**	29,2
Insuffisance cardiaque	6	12,5
Angor d’effort avec ischémie documentée	4	8,3
Ischémie silencieuse + ischémie documentée	2	4,2
Total	48	100,0

* dont 3 complications hémodynamiques dans les SCAST-

** dont 9 complications hémodynamiques et/ou rythmiques (choc cardiogénique, arrêt cardiaque,  ICG/OAP)

**Echocardiographie doppler et biologie**: La fraction d'éjection moyenne du ventricule gauche était de 48,17% ± 16,56 (extrêmes 22 et 76%). Elle était inférieure à 40% dans 29,2% des cas. Une insuffisance mitrale de grade I à III était retrouvée chez 35,4% des patients. Une hypertension artérielle pulmonaire était retrouvée chez 18,8% des patients. La troponine était élevée dans 60,4% des cas avec une moyenne de 5,33 ng/ml (extrêmes 0,08 et 62).

**Données de l'angiographie**: La voie d'abord était fémorale (91,6%) ou radiale (8,4%).

**Les lésions du tronc commun gauche**: Le tronc commun gauche (TCG) portait une lésion significative dans 100% des cas. Il y avait du thrombus angiographique dans 10,4% des cas. Dans 10,4% des cas (cinq cas) il s'agissait d'une dissection dont trois cas de dissection spontanée. Les lésions touchaient la distalité et/ou la bifurcation du TCG dans 77,1% des cas. L'ostium du tronc commun et le corps seul étaient touchés respectivement dans 20,8% et 2,1%. Les lésions du TCG portaientt sur deux sites dans 10,4%. Les lésions étaient majoritairement de type B (83,3%); rarement de type A ou C (8,3% chacun). Le pourcentage moyen de sténose du tronc commun était de 79,8% (extrêmes 50 et 100%).

**Les associations lésionnelles**: Les lésions du TCG étaient associées à une ou plusieurs lésions significative(s) du reste du réseau coronaire dans 93,8% des cas. Le nombre moyen de lésions associées était de 2,48 ± 1,77 (extrêmes 0 et 6). Elles étaient majoritairement pluritronculaires 64,6% dont tritronculaires (33,3%) et bitronculaires (31,3%). Le nombre moyen de troncs associés était de 2,06 ± 0,98. Le réseau de l'artère interventriculaire antérieure était touché dans 77,1% des cas, celui de l'artère circonflexe dans 68,8% des cas et celui de l'artère coronaire droite dans 43,8% des cas.

**Indications de la revascularisation**: La coronarographie a été réalisée en urgence dans 31,3% des cas et programmée dans 68,7% des cas. L'angioplastie ad'hoc a été réalisée dans 58,3% des cas. Le choix de l'angioplastie comme technique de revascularisation a été dicté par les critères décrits au Tableau 3. L'urgence (33,3%) et la contre-indication à la chirurgie de pontage (31,3%) ont été les principales indications. L'EuroSCORE moyen des patients était de 8,63 ± 4,11 (extrême 0 et 19). Il était supérieur ou égal à 6 dans 77,1% des cas et supérieur ou égal à 10 dans 37,5%.

**Tableau 3:** Critères de choix de l’angioplastie pour la revascularisation des lésions du tronc commun non protégé de l’artère coronaire gauche chez 48 patients

Critères de choix	Nombre de cas (n)	Pourcentage (%)
Urgence	16	33,3
Contre-indication à la chirurgie	15	31,3
Age avancé	6	12,5
Refus de la chirurgie	1	2,1
Anévrisme du tronc post dissection spontanée	1	2,1
Stent actif < 3-6 mois	2	4,2
Mauvais réseau d’aval	2	4,2
Autres raisons	5	10,4
**Total**	48	100,0

**Résultats de l'angioplastie**: Quarante-sept patients (97,9%) ont bénéficié d'une angioplastie avec stent. Il s'agissait d'un stent nu dans 72,9% des cas et d'un stent actif dans 25% des cas. Une angioplastie au ballon seule a été réalisée chez un patient (2,1%). Le stenting direct a été réalisé dans 72,9% des cas. Le stent était en direction de l'artère interventriculaire antérieure (IVA) dans 64,6% et vers la circonflexe dans 10,4%. Un kissing IVA-circonflexe a été réalisé dans 54,2% des cas. Le nombre moyen de lésions traitées était de 1,88 par patient (extrêmes 1 et 5). Le nombre de stents utilisés variait de 0 à 5 avec une moyenne de 1,81 stent par patient. La longueur moyenne de stent par patient était de 26,96 ± 21,30 mm (extrêmes 0 et 98). Un succès angiographique a été obtenu dans 97,9% avec un flux TIMI III sur le tronc et les principales branches d'aval. La revascularisation a été complète dans 58,3% des cas. Dix cas (20,8%) de complications ont été enregistrés durant la procédure. Il s'agissait de trois dissections coronaires, trois occlusions transitoires de coronaire d'aval, un SCAST+, une perforation du tronc par rotablator sur un tronc très calcifié et deux décès malgré le succès angiographique. Ces décès ont été enregistrés chez deux patients de 84 et 83 ans admis en état de choc cardiogénique avec respectivement une occlusion tritronculaire et une lésion monotronculaire ayant bénéficié d'un stenting IVA proximale trois mois plus tôt. Aucune complication cardiaque hospitalière post-angioplastie n'a été enregistrée. Sept complications extracardiaques (14,6%) post-angioplastie sont survenues: une thrombopénie au clopidogrel suppléé avec succès par la ticlopidine, deux hématomes au point de ponction artérielle avec faux anévrismes résolutifs sans intervention chirurgicale, une rectorragie sur diverticulose colique, deux surinfections de broncho-pneumopathie chronique obstructive et l'aggravation d'une insuffisance rénale chronique améliorée sans recours à la dialyse. La durée moyenne d'hospitalisation a été de 9,58 ± 6,2 jours (extrêmes 1 et 27). La durée moyenne de séjour après l'angioplastie a été de 6,73 ± 6,02 jours (extrêmes 2 et 26). Le taux de décès hospitalier a été de 4,2%. Pendant l'hospitalisation 22,9% des patients avaient été traités par les anti-GPIIb/IIIa, 10,4% avaient bénéficié d'une contre-pulsion par ballon intra-aortique et 14,6% avaient eu des amines vasopressives. Le traitement conventionnel optimal a été prescrit à la sortie de l'hôpital chez 58,8% des patients. Le taux de prescription des différentes familles varie de 80,4% pour les inhibiteurs de l'enzyme de conversion de l'angiotensine/antagonistes de récepteurs de l'angiotensine II à 100% pour la double antiagrégation plaquettaire.

**Evolution post hospitalière**: Le suivi post hospitalier moyen a été de 22,44 ± 20,08 mois (extrêmes 1 et 70 mois) avec une médiane de 17 mois. La double antiagrégation plaquettaire a duré en moyenne 17, 67 ± 17,62 mois. Le Tableau 4 résume l'évolution des patients après l'angioplastie. Au terme du suivi, le taux d'événements cardiovasculaires a été de 31,3%, le taux de revascularisation ultérieure de 16,6% dont 14,6% sur la lésion cible et le taux de décès toute cause de 31,3%. La cause du décès était cardiaque dans 53,3% des cas et mixte (cardiaque et rénale) dans 20% des cas. Les 2/3 des décès sont intervenus dans les dix premiers mois suivant l'angioplastie initiale. Parmi les survivants 90,9% étaient asymptomatiques et 9,1% avaient un angor d'effort. La deuxième revascularisation a été relaissée par stenting (50%), l'angioplastie transluminale au ballon seul (12,5%) et le pontage aorto-coronarien (37,5%). Aucune revascularisation n'a été réalisée en urgence. La revascularisation est intervenue dans les six premiers mois de l'angioplastie initiale pour six patients (75%). La revascularisation complète initiale a été un facteur prédictif de bonne évolution clinique et de survie (odds ratio de 0,08 (0,01 et 0,42; IC à 95%, P < 0,0003). Les facteurs de mauvais pronostic ont été un EuroSCORE ≥ 10 (OR = 5,4 [1,17 < OR < 26,63], P < 0,01; IC à 95%) et la présence d'antécédents cardiovasculaires et/ou de co-morbidités (OR = 10,11; P < 0,01; IC à 95%). La Fraction d'éjection ventriculaire gauche des patients décédés était significativement plus basse que celle des survivants (36,73 ± 17,02% contre 53,47 ± 13,87%).

**Tableau 4:** Evénements cardiovasculaires après angioplastie du tronc commun non protégé de l’artère coronairegauche chez 48 patients

Evénements	Intrahospitalier	A 30 jours Nombre (%)	A 6 moisNombre (%)	A 12 moisNombre (%)	Au terme de l’étudeNombre (%)
ECVM*	2 (4,2)	6 (12,5%)	12 (25)	12 (25)	15 (31,3)
Resténose	-	0	9 (18,8)	9 (18,8)	10 (20,8)
Revascularisation	0	0	6 (12,5)	7(14,6)	8 (16,7%)**
Décès	2 (4,2)	4 (8,4)	7 (14,6)	10(20,8)	15 (31,3)

*ECVM=évènements cardiovasculaires majeurs: Décès+IDM non fatal avec onde Q+Insuffisance cardiaque+revascularisation en urgence

**14,6% de revascularisation sur la lésion cible et une lésion de novo

## Discussion

Les principales indications de l'angioplastie du TCGNP dans notre étude étaient l'urgence ou la contre-indication à la chirurgie de pontage aorto-coronarien. Dans ce type d'indication il s'agit de patients à haut risque chirurgical ou cardio-vasculaire [5]. Dans notre étude 75% des patients étaient admis au décours d'un syndrome coronarien aigu (SCA) dont 29,2% pour SCAST+. Un tiers de ces SCA étaient compliqués de défaillance hémodynamique grave. Aux syndromes coronariens aigus il faut ajouter 12,5% de défaillance cardiaque congestive ou dœdème aigu pulmonaire inaugural en dehors des SCA. Valgimigli et al ont rapporté 50% de SCA ou avec fraction d'éjection ventriculaire gauche < 45% et 9% de défaillance cardiaque grave dans leur série pour 2/3 de pluritronculaires [6]. Les SCA et la défaillance cardiaque congestive ont été identifiés comme des facteurs prédictifs d'événements cardiovasculaires majeurs [5, 7-9]. Au plan angiographique les lésions du tronc commun coronaire gauche sont souvent complexes, calcifiées, distales englobant ou non la bifurcation IVA-circonflexe et nécessitant alors la réalisation d'un kissing [5]. Elles sont souvent associées à des lésions pluritronculaires [5, 10]. Notre étude confirme ces données avec 77,1% de lésions distales, 83,3% de lésions de type B; 8,3% de lésion de type C et 64,6% de lésions pluritronculaires. Dans 20,8% des cas un recours à l'échographie endocoronaire a été nécessaire pour une description plus précise de la lésion ou pour contrôler le résultat du stenting. Cette technique, aussi utilisée par d'autres équipes [9], devrait être utilisée de manière systématique pour s'assurer de l'absence de malposition de stent. Les taux de mortalité hospitalier (4,2%), à six mois (14,6%) et un an (20,8%) sont comparables aux données de la littérature y compris dans les études cliniques sélectionnant des patients moins graves [10, 11].

Le taux de revascularisation ultérieure est également comparable aux taux retrouvés dans la littérature. En effet dans une revue de la littérature Taggart et al. [11] ont rapporté des taux de décès hospitalier ou dans les 30 jours de 0 à 14% avec les stents nus. A deux ans il est de 3-31%. Le taux de revascularisation à 30 jours varie de 3 à 20% et de 15 à 34% à deux ans [11]. Dans notre étude aucune revascularisation ultérieure n'a été réalisée dans les 30 jours. Le taux de revascularisation après un suivi moyen de 22 mois a été de 16,7%. Dans une population non sélectionnée, avec un EuroSCORE très élevé (19 ± 23), Migliorini et al. retrouvaient des taux de décès global de 9,9% à un mois et de 50% pour les patients admis pour infarctus du myocarde chez des patients traités par angioplastie avec des stents actifs [5]. Ce taux était beaucoup plus élevé (21% à un mois) pour les patients avec un EuroSCORE ≥ 13. A 6 mois, le taux de mortalité globale était de 12,8% et le taux de resténose et de revascularisation de 16% [5]. Nous retrouvons un taux de resténose de 18,8% à six mois. Les données issues de SYNTAX [3] et les études de registres [8, 12, 13] ont montré que l'angioplastie du tronc commun avec stents actifs offre la même sécurité que le pontage à moyen terme. Le taux de mortalité est de 3,5 à 12% pour le groupe ponté contre 4,3 à 13% pour le groupe angioplastie après un suivi de 12 à 14 mois [3, 8]. Les taux de revascularisation ultérieure pour le pontage et l'angioplastie sont respectivement de 3 à 5,9% et de 13,7 à 26% [3, 8, 13]. Sur une série de 2240 patients avec un suivi médian de trois ans Seung et al [14] ne retrouvent pas de différence sur le taux de décès et le taux combiné de décès-infarctus du myocarde et accidents vasculaires cérébraux entre le pontage et l'angioplastie aussi bien avec stents actifs qu'avec stents nus avec cependant un taux de revascularisation ultérieure supérieur dans le groupe angioplastie. Notre étude, malgré ses limites (rétrospective, absence de groupe chirurgie et effectif relativement petit), retrouve un taux de revascularisation ultérieure de la lésion cible de 14,6% après un suivi moyen de 22 mois. Nos patients avaient reçu en majorité des stents nus (73%) dû au fait que beaucoup étaient en phase aiguë et à la non indication du stent actif sur le tronc commun en son temps.

Le taux de resténose au terme de l'étude a été de 20,8% mais le contrôle angiographique n'a été réalisé que chez 43,8% de nos patients. Le contrôle coronarographique systématique retrouve un taux de resténose important (34% à 3 mois et 44% à 9 mois) car les resténoses sont souvent asymptomatiques [15]. Notre taux a pu être influencé par la localisation distale prédominante englobant la bifurcation, l'utilisation prédominante des stents nus ainsi que le nombre de troncs et de lésions traités. Le siège distal des lésions du TCG, et les lésions de bifurcation en général sont reconnus comme des facteurs prédictifs d'événements coronariens majeurs chez les patients revascularisés par angioplastie [16, 17]. Il est aussi établi que le taux de resténose et d'événements cardiovasculaires majeurs est plus élevé avec les stents nus qu'avec les stents actifs [8, 14, 18-21] y compris sur le tronc commun [18, 22]. Le taux de survie de 68,8% n'est pas négligeable au regard de l'état clinique initial de ces patients et du risque spontané en l'absence de revascularisation. Les facteurs prédictifs d'évolution péjorative dans notre étude (décès, événements cardiovasculaires majeurs) sont un EuroSCORE ≥ 10, une fraction d'éjection ventriculaire basse et la présence d'antécédents et/ou de comorbidités (insuffisance rénale chronique sévère ou terminale, néoplasie). Dans la littérature les SCA, l'âge ≥ 70 ans, l'insuffisance cardiaque stade III-IV de la New York Heart Association et les artériopathies périphériques sont des facteurs prédictifs indépendants de mauvais pronostic [8].

## Conclusion

Cette étude a confirmé les données de la littérature. Même chez les patients non sélectionnés ou à risque opératoire élevé, l'angioplastie du tronc commun non protégé est réalisable. Le risque vital ou d'événements cardiovasculaires ultérieurs des patients est étroitement lié à leur risque avant l'angioplastie.

### Etat des connaissances actuelle sur le sujet

- La chirurgie de pontage aorto-coronarien est le traitement de référence des lésions du tronc commun coronaire gauche non protégé (TCGNP) car elle est mieux documentée et donne de meilleurs résultats à long termes et limite les revascularisations ultérieures;
- L'angioplastie est une alternative pour les patients non opérables ou avec gros risques opératoires;
- L'angioplastie avec stents et particulièrement les stents actifs donnent des résultats encourageants à court et moyen terme.

### Contribution de notre étude à la connaissance

- La place de l'angioplastie dans le traitement des lésions du TCGNP est évolutive avec le temps et les preuves apportées par les études;
- Notre étude contribue ainsi à l'ancrage de l'angioplastie dans le traitement des lésions du TCGNP.

### Conflits d’intérêts

Les auteurs ne déclarent aucun conflit d'intérêts.

## Remerciements

Tous nos remerciements aux Prs Jean-René Lusson, Pascal Motreff et Dr Géraud Souteyrand pour leur accueil et leurs contributions diverses pour la réalisation de ce travail. Tous non remerciements aux Prs Jean-René Lusson, Pascal Motreff et Dr Géraud Souteyrand pour leur accueil et leurs contributions diverses pour la réalisation de ce travail.
